# Geriatric Nutrition Risk Index: Prognostic factor related to inflammation in elderly patients with cancer cachexia

**DOI:** 10.1002/jcsm.12800

**Published:** 2021-09-29

**Authors:** Guo‐Tian Ruan, Qi Zhang, Xi Zhang, Meng Tang, Meng‐Meng Song, Xiao‐Wei Zhang, Xiang‐Rui Li, Kang‐Ping Zhang, Yi‐Zhong Ge, Ming Yang, Qin‐Qin Li, Yong‐Bing Chen, Kai‐Ying Yu, Ming‐Hua Cong, Wei Li, Kun‐Hua Wang, Han‐Ping Shi

**Affiliations:** ^1^ Department of Gastrointestinal Surgery/Department of Clinical Nutrition, Beijing Shijitan Hospital Capital Medical University Beijing China; ^2^ Department of Oncology Capital Medical University Beijing China; ^3^ Beijing International Science and Technology Cooperation Base for Cancer Metabolism and Nutrition Beijing China; ^4^ The Second Affiliated Hospital and Yuying Children's Hospital of Wenzhou Medical University Wenzhou China; ^5^ Comprehensive Oncology Department, National Cancer Center/Cancer Hospital Chinese Academy of Medical Sciences and Peking Union Medical College Beijing China; ^6^ Cancer Center, the First Hospital Jilin University Changchun China; ^7^ Department of Surgery The First Affiliated Hospital of Kunming Medical University Kunming China

**Keywords:** GNRI, Systemic inflammation, Cancer cachexia, Elderly, Overall survival

## Abstract

**Background:**

Systemic inflammation and cachexia are associated with adverse clinical outcomes in elderly patients with cancer. The Geriatric Nutritional Risk Index (GNRI) is a simple and useful tool to assess these conditions, but its predictive ability for elderly patients with cancer cachexia (EPCC) is unknown.

**Methods:**

This multicentre cohort study included 746 EPCC with an average age of 72.00 ± 5.24 years, of whom 489 (65.5%) were male. The patients were divided into two groups (high GNRI group ≥91.959 vs. low GNRI group <91.959) according to the optimal cut‐off value of the ROC curve. The calibration curves were performed to analyse the prognostic, predictive ability of GNRI. Comprehensive survival analyses were utilized to explore the relationship between GNRI and the overall survival (OS) of EPCC. Interaction analysis was used to investigate the comprehensive effects of low GNRI and subgroup parameters on the OS of EPCC.

**Results:**

In this study, a total of 2560 patients were diagnosed with cancer cachexia, including 746 cases of EPCC. During the 3.6 year median follow‐up, we observed 403 deaths. The overall mortality rate for EPCC at 12 months was 34.3% (95% CI: 62.3% to 69.2%), and resulting in rate of 278 events per 1000 patient‐years. The GNRI score of EPCC was significantly lower than those of young patients with cancer cachexia (*P* < 0.001). The 1, 3, and 5 year calibration curves showed that the GNRI score had good survival prediction in the OS of EPCC. The GNRI could predict the OS of EPCC, whether as a continuous variable or a categorical variable. Particularly, we also found that low GNRI score (<91.959) of EPCC had a worse prognosis than those with a high GNRI score (≥91.959, *P* = 0.001, HR = 1.728, 95% CI: 1.244–2.401). Consistent results were observed in the tumour subgroups of gastric cancer and colorectal cancer. Notably, similar results were observed in the sensitivity analysis. In the subgroup analysis, the low GNRI has a combined effect with age (<70 years) on poor OS of EPCC. The results of the prognostic risk model found that the lower the GNRI score, the greater the prognostic risk score, and the greater the risk of death in EPCC.

**Conclusions:**

For the first time, this study found that the GNRI score can serve as an independent prognostic factor for the OS of EPCC.

## Introduction

Recently, the International Agency for Research on Cancer (IARC) of the World Health Organization released the latest global cancer burden data in 2020. According to reports, in 2020, there will be 19.29 million new cancer cases worldwide, of which 10.06 million are men and 9.23 million are women. Importantly, there are 9.96 million cancer‐related deaths, including 5.53 million men and 4.43 million women. As far as China is concerned, the number of new cancer‐related cases and deaths ranks first globally.^1^ In 2021, the USA is expected to add 1 898 160 new cancer cases and 608 570 cancer deaths.^2^ The global burden of cancer is getting heavier. Cachexia is considered to be the leading cause of death in cancer patients, with high morbidity and mortality.^3^ Cancer cachexia is a syndrome characterized by weight loss, adipose tissue consumption, and decreased muscle mass. It can lead to impaired body functions, immune response, physical performance, decreased quality of life, reduced response to treatment, reduced treatment tolerance, and poor prognosis.^4^, ^5^, ^6^ Cachexia can occur in the context of a variety of tumour types, but it is most common in upper gastrointestinal (GI) cancer and lung cancer, among which 83% of pancreatic and gastric cancer patients develop cachexia, and 60% of lung cancer patients develop cachexia.^7^

Aging is an inevitable worldwide problem for humankind. Older adults are one of the most heterogeneous and vulnerable groups, and they face a higher risk of nutritional problems.^8^ Compounding the problem is that older adults are more susceptible to cancer and more likely to suffer from cancer cachexia.^9^, ^10^ Up to 65% of patients referred to specialized geriatric oncology clinics for geriatric evaluation were found to have cancer cachexia.^4^ All patients with cachexia suffer from malnutrition, but cachexia is not always present in all patients with malnutrition.^11^ Unlike starvation and malnutrition patients who can be easily reversed by diet intervention,^12^ and aging is related to the progressive reduction of systemic protein and impaired ability to cope with physiological stress, the treatment of elderly patients with cancer cachexia (EPCC) will be a long‐term process.^13^ Importantly, when compared with younger patients, elderly patients have a higher prognosis risk, and normal organ functions also decline with aging. Although many indicators have been reported on the role of EPCC, there is a lack of a targeted elderly related indicator to predict the prognosis of EPCC. Therefore, it is extremely urgent to find a suitable and practical prognostic detection tool in old adults with cancer cachexia.

Considering these concerns, we evaluated the prognostic monitoring value of the geriatric nutritional risk index (GNRI) in EPCC. This is a newly proposed, simple, and objective method to assess the nutritional status of elderly patients.^14^ The GNRI, which is a modified version of the nutritional risk index (NRI), can be easily calculated from routine haematology data (serum albumin) and anthropometric measurements (including height and weight). These indicators are easy to obtain, especially weight and height, which can reduce information bias.^14^ Both serum albumin concentration and BMI are important parameters that can reflect the risk of survival in patients with malignancies. Albumin itself is a sign of inflammation and can reflect the severity of the acute disease.^15^ On the contrary, the body mass index (BMI) is a reasonable indicator of obesity. Elevated BMI is a risk factor for cancer death.^16^ Notably, the prognostic prediction ability of GNRI is superior to BMI and serum albumin levels alone.^17^ Previous studies have emphasized the usefulness of GNRI in assessing the physical health of elderly patients with chronic diseases.^18^ Additionally, GNRI can reflect the nutritional status and system inflammation of old adults with cancer.^19^ The accumulated evidence supports the harmful effects of malnutrition of various malignant tumours on patients' survival time.^20^ Recent studies have evaluated the role of GNRI in elderly patients with various cancers, including lung cancer,^19^ prostate cancer,^21^ head and neck cancer,^22^ and gastrointestinal cancer.^23^, ^24^, ^25^ However, the prognostic value of GNRI in EPCC has not been thoroughly clarified, so this study aims to investigate the potential prognostic value and clinical outcome prediction ability of GNRI in EPCC.

## Materials and methods

### Patients and study design

This multi‐centre cohort study recruited a total of 12 792 patients with cancer from multiple regional central hospitals in China from June 2012 to December 2019. In this multi‐centre study, the inclusion criteria were (i) age ≥18 years; (ii) hospital stays longer than 48 h; (iii) pathological diagnosis of cancer; (iv) patients without serious infection and immunodeficiency syndrome and other serious diseases; and (v) signed informed consent. The studies that met any of the following were excluded: (i) under 18 years of age; (ii) hospital stay <48 h; (iii) patients with serious infections and immunodeficiency syndrome and other serious diseases; and (iv) refusal to sign informed consent. After removing some missing data information, a total of 9728 cancer patient parameter information was obtained, of which 2560 were cancer cachexia patients. Finally, patients with cancer cachexia more than 65 years old were included in this cohort, and there was a total of 746 EPCC patients with complete clinical parameter information.^26^ The detailed flow chart is shown in the Supporting Information, *Figure* S1. This study complied with the Declaration of Helsinki and was approved by the institutional ethics committees of all participating institutions. All participants signed an informed consent form (Registration number: ChiCTR1800020329).

### Evaluation and definition

We collected information on demographic information, clinical parameter information, physical measurements, and laboratory tests for all participants, including age, sex, height, weight, site of cancer [lung cancer (LC), gastrointestinal cancer (gastric cancer, GC; colorectal cancer, CRC; oesophageal cancer, EC; other gastrointestinal cancer), and other cancer subtypes], family history of cancer, co‐morbidities (diabetes, hypertension, and coronary heart disease), life and eating habits (smoking, tea consumption, and alcohol consumption), tumour node metastasis (TNM) stage, radical resection, postoperative chemoradiotherapy, Karnofsky Performance Status (KPS), Eastern Cooperative Oncology Group Performance Status (ECOG PS) Physical activity, nutritional intervention, and laboratory measurement indicators [white blood cells (WBC), lymphocytes, neutrophils, platelet, haemoglobin, aspertate aminotransferase (AST), alanine transaminase (ALT), serum total protein, serum albumin]. Additionally, we obtained calculated variables based on the information of these variables, namely, body mass index (BMI), Prognostic Nutritional Index (PNI), and Geriatric Nutrition Risk Index (GNRI). BMI was calculated as follows: BMI (kg/m^2^) = weight/height^2^. PNI was calculated using the following equation: PNI = 10 × albumin (g/dL) + 0.005 × lymphocytes count (/mm^3^). GNRI was calculated using the formula: GNRI = 14.89 × serum albumin (g/dL) + 41.7 × [present body weight (kg)/ideal body weight (kg)]. The ideal weight was defined as [height (m)]^2^ × 22. TNM staging is based on the eighth edition of the AJCC TNM classification system. The BMI classification standard refers to the Asian BMI classification standard. The cut‐off values of PNI and GNRI are based on the best cut‐off value of the ROC curve drawn by the R platform. At baseline, anthropometric measurements were performed by well‐trained staff. Laboratory indicators were sent to the laboratory for professional testing per hospital standards.

### Assessment of cancer cachexia

The definition and diagnosis of cancer cachexia were followed by Fearon criteria^6^: (i) unintentional weight loss of more than 5% in the past 6 months; (ii) BMI < 20 kg/m^2^ and any degree of weight loss >2%; (iii) skeletal muscle mass (sarcopenia) and any degree of weight loss >2%. The skeletal muscle depletion was assessed as follows: mid upper‐arm muscle area by anthropometry (men <32 cm^2^, women <18 cm^2^).

### Outcome evaluation

All patient follow‐up information was obtained through regular outpatient follow‐up or telephone. The Kaplan–Meier method was used to analyse the overall survival (OS), and the log‐rank test was used for comparison. Multivariate Cox regression analysis was used to determine independent predictors of OS. OS is determined from the date of the first diagnosis of cancer to the date of death, the date of withdrawal from the study, or the time of the last follow‐up.

### Statistical analysis

In the baseline presentation, continuous variables were expressed as mean ± standard deviation (SD), while categorical variables were reported as the number of patients (percentage, %). The comparison between continuous variables used the Student's *t*‐test and the comparison between categorical variables used the *χ* ^2^ test. If the data do not conform to the normal distribution, the nonparametric test was used for statistical analysis. Pearson correlation analysis was utilized to analyse the correlation between GNRI and related variables, and restricted cubic spline regression was performed to investigate the prognostic relationship between GNRI and OS. The calibration curve analysis was used to assess the prognostic predictive ability of GNRI.

Univariate and multivariate Cox regression analyses were performed to analyse the independent prognostic value of GNRI in the OS of patients with cancer cachexia. All statistical analysis in this study is done by R software [version 4.0.3 (Bunny‐Wunnies Freak Out), https://www.r‐project.org/]. All two‐tailed statistical *P* values <0.05 were considered statistically different.

## Results

### Baseline data comparison based on Geriatric Nutritional Risk Index

**Figure 1:**
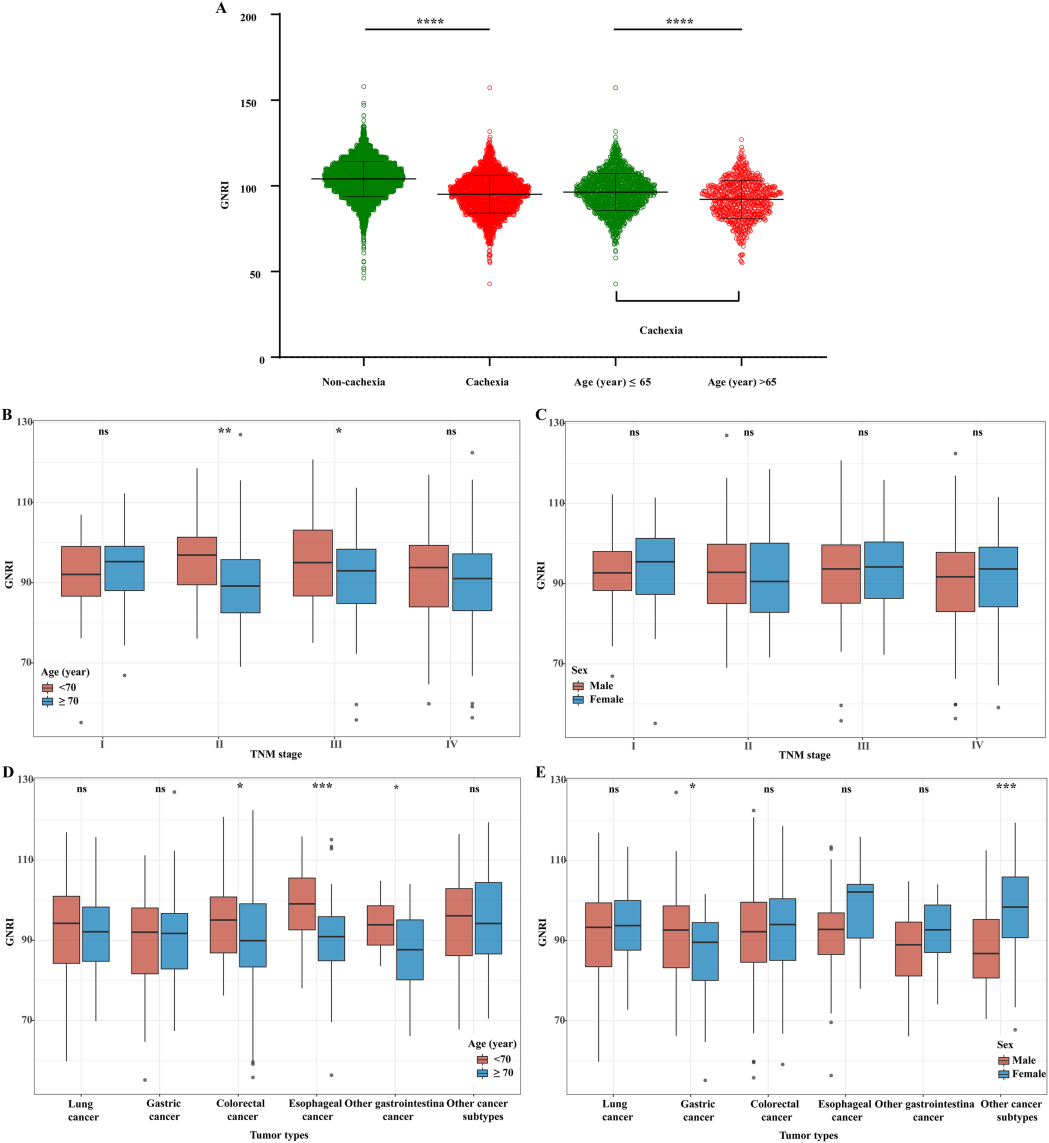
The distribution of GNRI in different groups. (A) The distribution of GNRI in cachexia and non‐cachexia population and in different age groups. (B–E) The distribution of GNRI in different EPCC. The distribution of GNRI in different TNM stages based on age subgroup (B) and sex subgroup (C); the distribution of GNRI in different tumour types based on age subgroup (D) and sex subgroup (E). EPCC, Elderly Patients with Cancer Cachexia; GNRI, Geriatric Nutritional Risk Index.

In this study, a total of 9728 patients with cancer were included in the cohort, and 2560 (26.3%) cases of patients were diagnosed with cancer cachexia, including 746 cases of EPCC with an average age of 72.00 ± 5.24 years, of whom 489 (65.5%) were male. We compared the GNRI scores of the cachexia population and the non‐cachexia population in the cancer cohort and found that the GNRI scores of non‐cachexia cancer patients were significantly higher than those of cancer cachexia patients (*P* < 0.001). We compared the GNRI scores of young and EPCC and found that the GNRI scores of EPCC were significantly lower than those of young patients with cancer cachexia (*P* < 0.001). Additionally, we also analysed the distribution of GNRI scores in patients with different TNM stages and different cancer types in the age (<70 years vs. ≥70 years) and sex (male vs. female) of the EPCC. The distribution of GNRI scores in different TNM stages showed that the GNRI scores of Stage II and III in older patients (age ≥70 years) was significantly lower than those patients with age <70 years (*P* < 0.01). In the analysis of the distribution of GNRI scores in different cancers, it was found that in the main tumour subgroups, the GNRI score of GC in male patients was significantly higher than that of female patients (*P* < 0.05), and the GNRI scores of CRC and EC in older patients (age ≥ 70 years) were significantly lower than those of patients aged <70 years (*P* < 0.05 and *P* < 0.001, respectively) (*Figure* 1.)

**Figure 2:**
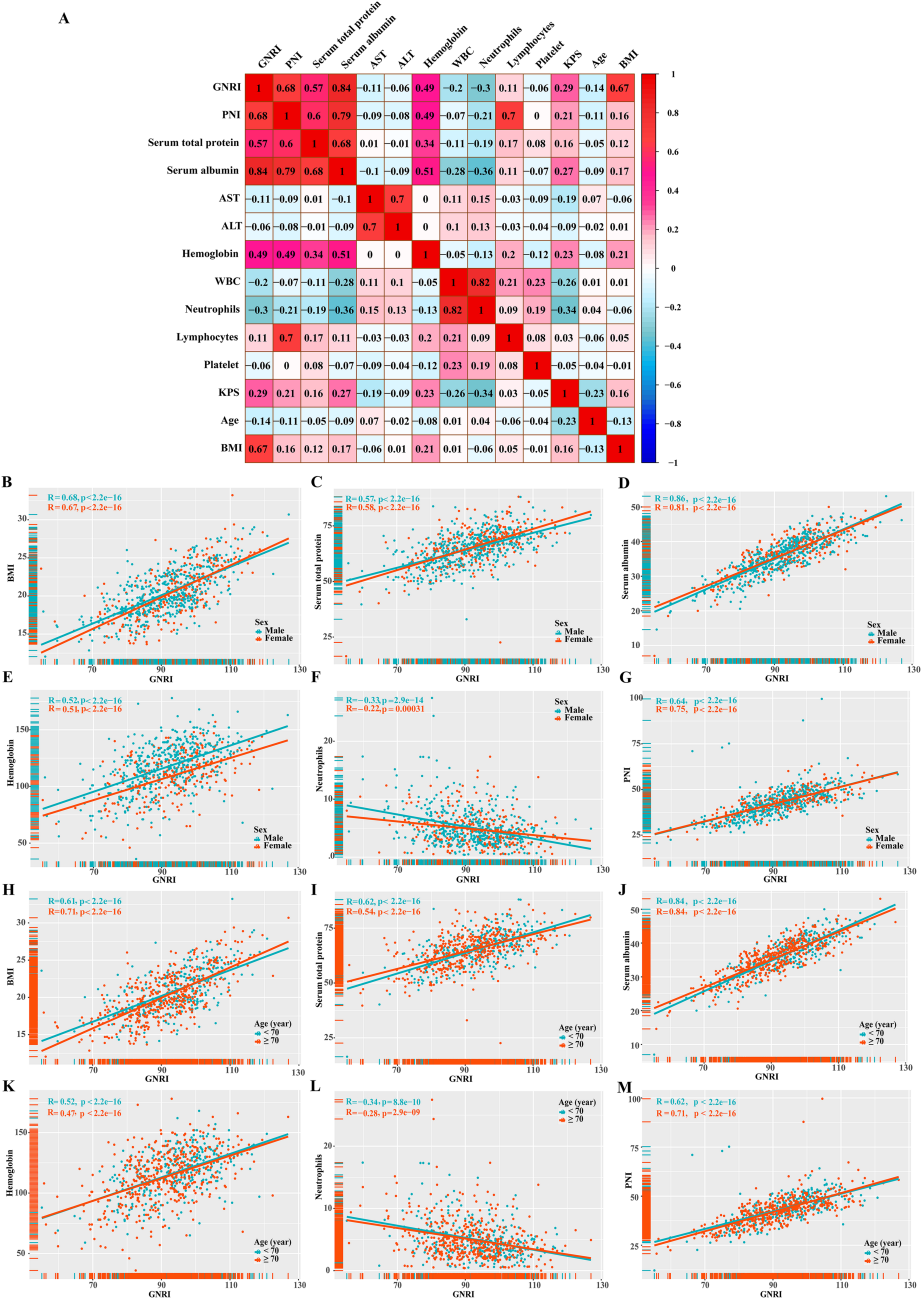
The Person analysis of GNRI and related‐factors. (A) Overall patients; sex subgroup for BMI (B), serum total protein (C), serum albumin (D), haemoglobin (E), neutrophil count (F), and PNI (G); age subgroup for BMI (H), serum total protein (I), serum albumin (J), haemoglobin (K), neutrophil count (L), and PNI (M). BMI, body mass index; GNRI, Geriatric Nutritional Risk Index; PNI, Prognostic Nutritional Index.

We conducted a Pearson analysis of GNRI and different clinically relevant parameters on EPCC; the consequence found that GNRI was significantly correlated with BMI, serum total protein, serum albumin, haemoglobin, neutrophil count, and PNI (*R* > 0.3 or *R* < −0.3, *P* < 0.05). Then, based on the results of Pearson analysis, we did a stratified Pearson analysis at different ages (≥70 years old vs. <70 years old) and gender (male vs. female), GNRI and BMI, serum total protein, serum albumin, haemoglobin, and PNI are positively correlated in different ages and genders. When analysing the stratification of the relationship between GNRI and neutrophil count, the results showed that male patients and patients <70 years old had a significant negative correlation with GNRI (R < −0.3, *P* < 0.05) (*Figure* 2).

### Patient characteristics stratified by Geriatric Nutritional Risk Index

Over a median of 3.7 years of follow‐up, we observed 403 deaths. The overall mortality rate for EPCC at 12 months was 34.3% (95% CI: 62.3% to 69.2%), and resulting in rate of 278 events per 1000 patient‐years. The grouping of GNRI and PNI was determined based on the best cut‐off value of ROC. The cut‐off values of GNRI and PNI were 91.959 and 42.425, respectively. According to the cut‐off value of GNRI, the patient was divided into high and low groups, namely, high score group (GNRI ≥ 91.959) and low score group (GNRI < 91.959). Based on the cut‐off value of PNI, the patient was divided into high score group (PNI ≥ 42.425) and low score group (PNI < 42.425) (Supporting Information, *Figure* S2).

At baseline, the average age of EPCC in this study was 72 ± 5.24 years (ranged from 66 to 95 years), 489 male patients (65.55%), and 257 female patients (34.5%). Among the main types of cancer, there were 162 cases of LC (21.7%), 165 cases of GC (22.1%), 201 cases of CRC (26.9%), and 95 cases of EC (12.7%). As shown in the GNRI stratification results, there were 396 patients in the high GNRI group and 350 patients in the low GNRI group. Difference analysis results showed age (*P* < 0.001), hypertension (*P* = 0.002), BMI (*P* < 0.001), ECOG PS (*P* < 0.001), KPS (*P* < 0.001), physical activity (*P* < 0.001), serum total protein (*P* < 0.001), serum albumin (*P* < 0.001), AST (*P* = 0.004), ALT (*P* = 0.0019), haemoglobin (*P* < 0.001), WBC (*P* < 0.001), neutrophils (*P* < 0.001), lymphocytes (*P* = 0.002), and PNI (*P* < 0.001) has different significance in the GNRI grouping population (*Table* 1).

**Table 1:** Characteristics of overall patients and stratified by GNRI

	Overall	Stratified by GNRI		
	Patients (*n*, %)	Low (<91.959)	High (≥91.959)	*P* value
Characteristics	(*n* = 746)	(*n* = 350)	(*n* = 396)	
Age (years), mean (SD)				<0.001
	72.00 (5.24)	72.75 (5.64)	71.33 (4.77)	
Sex, *n* (%)				0.087
Male	489 (65.5)	241 (68.9)	248 (62.6)	
Female	257 (34.5)	109 (31.1)	148 (37.4)	
Sites of cancer, *n* (%)				0.432
Lung cancer, *n* (%)	162 (21.7)	74 (21.1)	88 (22.2)	
Gastrointestinal cancer, *n* (%)	506 (67.8)	243 (69.4)	263 (66.4)	
Gastric cancer, *n* (%)	165 (22.1)	84 (24.0)	81(20.5)	
Colorectal cancer, *n* (%)	201(26.9)	93(26.6)	108(27.3)	
Esophageal cancer, *n* (%)	95 (12.7)	40 (11.4)	55 (13.9)	
Other gastrointestinal cancer, *n* (%)	45 (6.0)	26 (7.4)	19 (4.8)	
Other cancer subtypes, *n* (%)	78 (10.5)	33 (9.4)	45 (11.4)	
Diabetes, yes, *n* (%)	98 (13.1)	38 (10.9)	60 (15.2)	0.104
Hypertension, yes, *n* (%)	192 (25.7)	71 (20.3)	121 (30.6)	0.002
Coronary heart disease, yes, *n* (%)	70 (9.4)	28 (8.0)	42 (10.6)	0.275
Family history of cancer, yes, *n* (%)	94 (12.6)	40 (11.4)	54 (13.6)	0.426
Smoking, yes, *n* (%)	370 (49.6)	185 (52.9)	185 (46.7)	0.109
Alcohol consumption, yes, *n* (%)	160 (21.4)	73 (20.9)	87 (22.0)	0.779
Tea consumption, *n* (%)	198 (26.5)	98 (28.0)	100 (25.3)	0.444
BMI (kg/m^2^), mean (SD)	20.41 (3.17)	18.61 (2.44)	22.01 (2.88)	<0.001
TNM stage, *n* (%)				0.109
I	50 (6.7)	18 (5.1)	32 (8.1)	
II	159 (21.3)	71 (20.3)	88 (22.2)	
III	200 (26.8)	88 (25.1)	112 (28.3)	
IV	337 (45.2)	173 (49.4)	164 (41.4)	
Radical resection, yes, *n* (%)	215 (28.8)	96 (27.4)	119 (30.1)	0.479
Postoperative chemoradiotherapy, yes, *n* (%)	325 (43.6)	149 (42.6)	176 (44.4)	0.659
ECOG PS, *n* (%)				<0.001
<2	366 (49.1)	135 (38.6)	231 (58.3)	
≥2	380 (50.9)	215 (61.4)	165 (41.7)	
KPS, mean (SD)	79.26 (17.76)	75.20 (19.95)	82.85 (14.69)	<0.001
Physical activity				<0.001
Normal	499 (66.9)	203 (58.0)	296 (74.7)	
Limited	205 (27.5)	117 (33.4)	88 (22.2)	
Inactivity	42 (5.6)	30 (8.6)	12 (3.0)	
Nutritional intervention, yes, *n* (%)	174 (23.3)	90 (25.7)	84 (21.2)	0.172
Serum total protein (g/L), mean (SD)	65.01 (8.07)	61.17 (7.87)	68.40 (6.60)	<0.001
Serum albumin (g/L), mean (SD)	35.83 (5.55)	31.82 (4.50)	39.38 (3.67)	<0.001
AST (U/L), mean (SD)	31.26 (40.02)	35.71 (50.24)	27.33 (27.53)	0.004
ALT (U/L), mean (SD)	26.65 (30.98)	29.48 (37.46)	24.15 (23.59)	0.019
Haemoglobin (g/L), mean (SD)	114.49 (21.22)	105.25 (21.13)	122.65 (17.68)	<0.001
WBC (×10^9^/L), mean (SD)	7.29 (3.96)	7.92 (4.22)	6.74 (3.64)	<0.001
Neutrophils (×10^9^/L), mean (SD)	5.02 (3.26)	5.76 (3.80)	4.36 (2.51)	<0.001
Lymphocytes (×10^9^/L), mean (SD)	1.42 (0.95)	1.31 (0.95)	1.52 (0.94)	0.002
Platelet (×10^9^/L), mean (SD)	224.58 (95.23)	231.27 (103.96)	218.66 (86.49)	0.071
PNI, *n* (%)				<0.001
Low (<42.425)	339 (45.4)	272 (77.7)	67 (16.9)	
High (≥42.425)	407 (54.6)	78 (22.3)	329 (83.1)	

ALT, alanine transaminase; AST, aspertate aminotransferase; BMI, body mass index; ECOG PS, Eastern Cooperative Oncology Group Performance Status; GNRI, Geriatric Nutritional Risk Index; KPS, Karnofsky Performance Status; OS, overall survival; PNI, Prognostic Nutritional Index; WBC, white blood cells.

### Survival outcomes according to Geriatric Nutritional Risk Index

**Figure 3:**
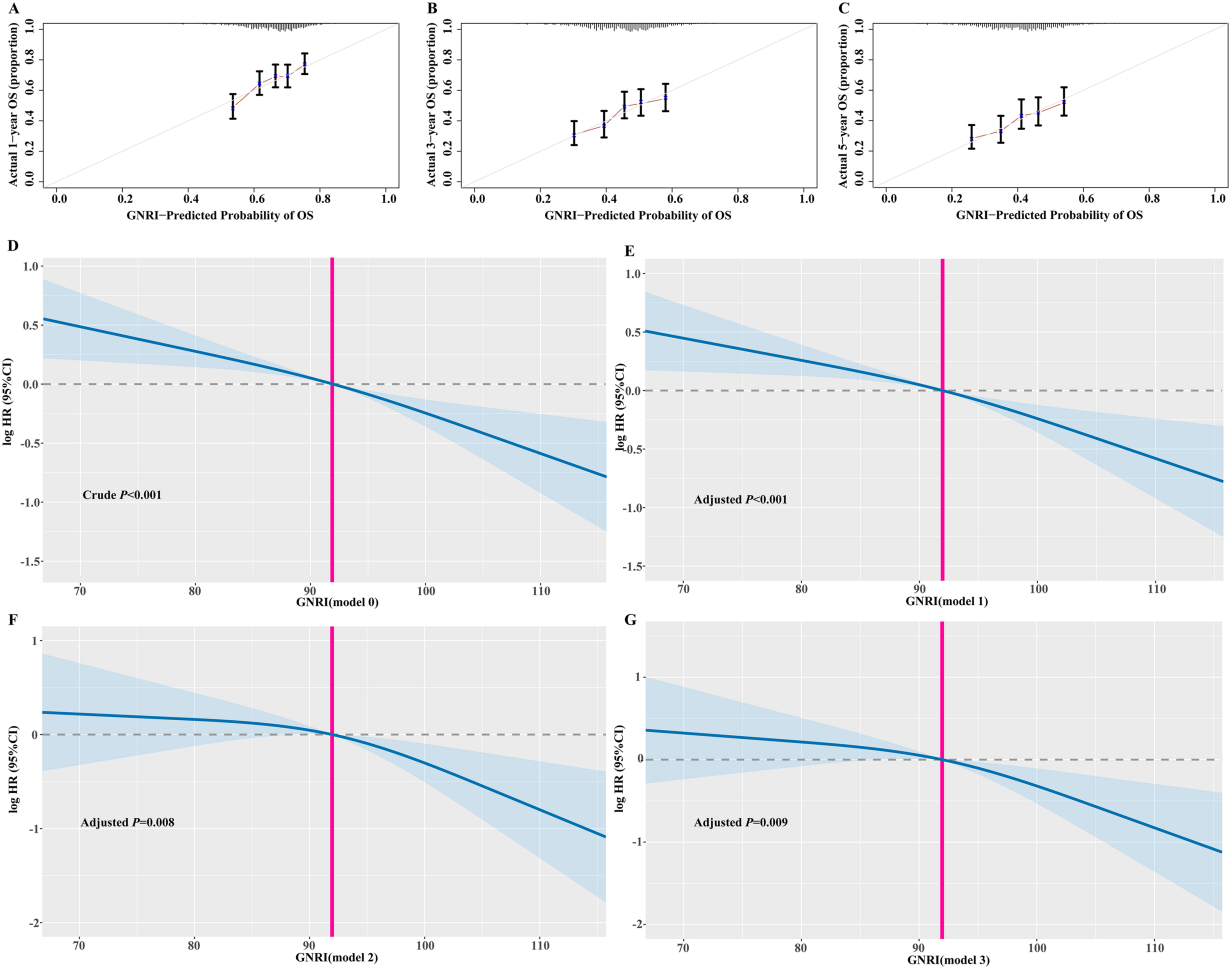
The calibration curves of GNRI and the association between GNRI and death risk of OS in EPCC. (A–C) 1, 3, and 5 year calibration curves of GNRI in EPCC; (D–G) the association between GNRI and death risk of OS in EPCC adjusted by different models. EPCC, Elderly Patients with Cancer Cachexia; GNRI, Geriatric Nutritional Risk Index; OS, overall survival.

We explored the prognostic value of GNRI in EPCC. The 1, 3, and 5 year calibration curve results showed that the GNRI score had a good survival prediction in the OS of EPCC (*Figure* 3A–C).

**Figure 4:**
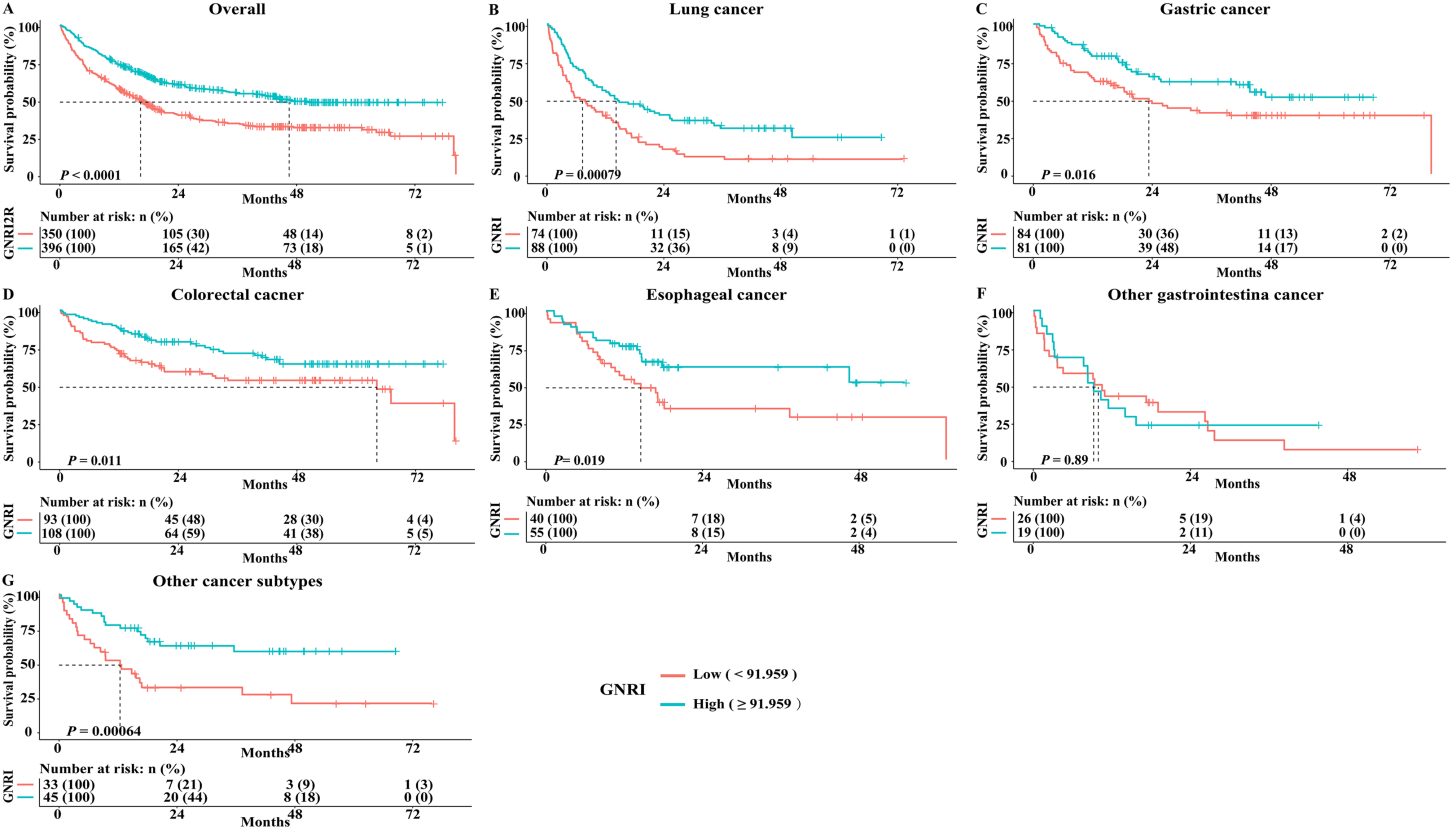
The Kaplan–Meier survival curves of GNRI in the OS of overall and cancer subtypes for EPCC. (A) Overall patients; (B) lung cancer; (C) gastric cancer; (D) colorectal cancer; (E) esophageal cancer; (F) other gastrointestinal cancer; (G) other cancer subtypes. EPCC, Elderly Patients with Cancer Cachexia; GNRI, Geriatric Nutritional Risk Index; OS, overall survival.

The Kaplan–Meier survival curve results indicated that the survival time of EPCC in the high GNRI score group was longer than that in the low GNRI score group (*P* < 0.001). This result was consistent in patients with LC, GC, CRC, EC, and other cancer types (all *P* < 0.05) (*Figure* 4). We performed univariate and multivariate survival analyses on clinical parameters. In univariate survival analysis, age, sex, ECOG, radical resection, TNM stage, KPS, postoperative chemoradiotherapy, lymphocytes, neutrophils, WBC, AST, ALT, serum albumin, and PNI were associated with the OS of EPCC (all *P* < 0.05). In the multivariate survival analysis, we analysed all clinical parameters (including GNRI) and found that tea consumption (yes vs. no; *P* = 0.032, HR = 0.762, 95% CI: 0.593–0.978), radical resection (yes vs. no; *P* = 0.001, HR = 0.563, 95% CI: 0.397–0.798), TNM stage (Stage III vs. Stage I; *P* = 0.044, HR = 1.856, 95% CI: 1.018–3.384; Stage IV vs. Stage I; *P* < 0.001, HR = 4.580, 95% CI: 2.455–8.541), KPS (50–80 and <50 vs. ≥80; *P* < 0.001, HR = 1.673, 95% CI: 1.265–2.213 and *P* = 0.011, HR = 1.873, 95% CI: 1.156–3.036), serum albumin (≥35 vs. <35; *P* = 0.036, HR = 1.407, 95% CI: 1.022–1.937), ALT (>50 vs. ≤50, *P* = 0.005, HR = 1.808, 95% CI: 1.195–2.736), and PNI (<42.425 vs. ≥42.425, *P* = 0.004, HR = 1.687, 95% CI: 1.181–2.411) were associated with the OS of EPCC (Supporting Information, *Table* S1).

Additionally, we investigated the prognostic value of GNRI in overall EPCC and different cancer types of EPCC. We constructed different adjustment models to reduce clinical bias, namely, Model 0: unadjusted; Model 1: adjusted for age, sex, and TNM stage; Model 2: adjusted for age, sex, ECOG, radical resection, TNM stage, KPS, physical activity, postoperative chemoradiotherapy, lymphocytes, neutrophils, WBC, AST, ALT, serum albumin, and PNI; Model 3: adjusted for age, sex, ECOG, radical resection, TNM stage, KPS, physical activity, nutritional intervention, postoperative chemoradiotherapy, lymphocytes, neutrophils, WBC, AST, ALT, serum albumin, PNI, family history of cancer, tea consumption, alcohol consumption, smoking, diabetes, hypertension, coronary heart disease, platelet, haemoglobin, and serum total protein. In overall patients, continuous GNRI was associated with better prognosis of OS in EPCC (Model 0: *P* < 0.001, HR = 0.759, 95% CI: 0.693–0.832; Model 1: *P* < 0.001, HR = 0.764, 95% CI: 0.696–0.838; Model 2: *P* = 0.030, HR = 0.833, 95% CI: 0.706–0.982; Model 3: *P* = 0.010, HR = 0.794, 95% CI: 0.665–0.948). When GNRI was used as a categorical variable, patients with low GNRI increased the death risk of OS in EPCC when compared with those patients with high GNRI (Model 0: *P* < 0.001, HR = 1.790, 95% CI: 1.468–2.181; Model 1: *P* < 0.001, HR = 1.782, 95% CI: 1.458–2.178; Model 2: *P* = 0.005, HR = 1.605, 95% CI: 1.158–2.224; Model 3: *P* = 0.001, HR = 1.728, 95% CI: 1.244–2.401). Consistently, when GNRI was classified into quartiles (Q1: >99.557, Q2: 92.922–99.557, Q3: 84.521–92.922, Q4: <84.521), patients with GNRI of Q3 (Model 3: *P* = 0.002, HR = 1.916, 95% CI: 1.262–2.908) and Q4 (Model 3: *P* = 0.003, HR = 2.339, 95% CI: 1.329–4.116) were significantly correlated with worse prognosis compared with Q1 group. In different types of cancer, after different adjustment models, GNRI was still an independent risk factor for OS in EPCC of GC and CRC. When compared with patients with high GNRI, low GNRI increased the risk of death in EPCC of GC (Model 0: *P* = 0.018, HR = 1.721, 95% CI: 1.098–2.698; Model 1: *P* = 0.029, HR = 1.660, 95% CI: 1.052–2.620; Model 2: *P* = 0.027, HR = 2.267, 95% CI: 1.099–4.673; Model 3: *P* = 0.016, HR = 2.460, 95% CI: 1.130–5.355) and CRC (Model 0: *P* = 0.013, HR = 1.783, 95% CI: 1.132–2.808; Model 1: *P* = 0.004, HR = 1.986, 95% CI: 1.244–3.170; Model 2: *P* = 0.052, HR = 2.290, 95% CI: 0.992–5.284; Model 3: *P* = 0.009, HR = 3.109, 95% CI: 1.333–7.253) (*Table* 2). The association between GNRI and death risk of OS in EPCC was showed in *Figure* 3D–G.

**Table 2:** Univariate and multivariate analysis on the OS of GNRI in overall cancer patients and different types of cancer patients

Variables	OS (model 0)		OS (model 1)		OS (model 2)		OS (model 3)		
	Crude HR (95% CI)	Crude *P*	Adjusted HR (95% CI)	Adjusted *P*	Adjusted HR (95% CI)	Adjusted *P*	Adjusted HR (95% CI)	Adjusted *P*	
Overall patients								
As continuous (per SD)	0.759 (0.693–0.832)	<0.001	0.764 (0.696–0.838)	<0.001	0.833 (0.706–0.982)	0.030	0.794 (0.665–0.948)	0.010
By GNRI cut‐off								
GNRI ≥91.959	1		1		1		1	
GNRI <91.959	1.790 (1.468–2.181)	<0.001	1.782 (1.458–2.178)	<0.001	1.605 (1.158–2.224)	0.005	1.728 (1.244–2.401)	0.001
By GNRI cut‐off								
GNRI <91.959	1				1		1	
GNRI ≥91.959	0.559 (0.458–0.681)	<0.001	0.561 (0.459–0.686)	<0.001	0.623 (0.450–0.864)	0.005	0.579 (0.417–0.804)	0.001
By Interquartile								
Q1 (99.557~)	1		1		1		1	
Q2 (92.922–99.557)	1.187 (0.881–1.600)	0.259	1.144 (0.849–1.542)	0.377	1.193 (0.844–1.686)	0.317	1.155 (0.814–1.637)	0.420
Q3 (84.521–92.922)	1.714 (1.319–2.228)	<0.001	1.685 (1.295–2.194)	<0.001	1.816 (1.201–2.746)	0.005	1.916 (1.262–2.908)	0.002
Q4 (~84.521)	2.271 (1.708–3.018)	<0.001	2.273 (1.703–3.034)	<0.001	2.061 (1.182–3.594)	0.011	2.339 (1.329–4.116)	0.003
*P* for trends		<0.001		<0.001		0.005		0.002
By tumour types								
Lung cancer								
GNRI ≥91.959	1		1		1		1	
GNRI <91.959	1.821 (1.277–2.597)	0.001	1.862 (1.296–2.675)	0.001	1.377 (0.750–2.531)	0.302	1.450 (0.755–2.783)	0.264
Gastrointestinal cancer								
Gastric cancer								
GNRI ≥91.959	1		1		1		1	
GNRI <91.959	1.721 (1.098–2.698)	0.018	1.660 (1.052–2.620)	0.029	2.267 (1.099–4.673)	0.027	2.460 (1.130–5.355)	0.023
Colorectal cancer								
GNRI ≥91.959	1		1		1		1	
GNRI <91.959	1.783 (1.132–2.808)	0.013	1.986 (1.244–3.170)	0.004	2.290 (0.992–5.284)	0.052	3.109 (1.333–7.253)	0.009
Esophageal cancer								
GNRI ≥91.959	1		1		1		1	
GNRI <91.959	2.014 (1.108–3.660)	0.022	1.802 (0.955–3.403)	0.069	0.625 (0.177–2.203)	0.464	1.396 (0.327–5.956)	0.652
Other gastrointestinal cancer								
GNRI ≥91.959	1		1		1		1	
GNRI <91.959	0.954 (0.481–1.893)	0.893	0.923 (0.369–2.311)	0.864	2.401 (0.250–23.021)	0.448	7.871 (0.251–247.231)	0.241
Other cancer subtypes								
GNRI ≥91.959	1		1		1		1	
GNRI <91.959	2.837 (1.517–5.303)	0.001	1.937 (0.987–3.800)	0.055	0.822 (0.175–3.874)	0.805	1.151 (0.216–6.146)	0.869

ALT, alanine transaminase; AST, aspertate aminotransferase; BMI, body mass index; CI, confidence interval; ECOG PS, Eastern Cooperative Oncology Group Performance Status; GNRI, Geriatric Nutritional Risk Index; HR, hazards ratio; KPS, Karnofsky Performance Status; OS, overall survival; PNI, Prognostic Nutritional Index; WBC, white blood cells.

Model 0: unadjusted. Model 1, adjusted for age, sex, and TNM stage. Model 2, adjusted for age, sex, ECOG, radical resection, TNM stage, KPS, physical activity, postoperative chemoradiotherapy, lymphocytes, neutrophils, WBC, AST, ALT, serum albumin, and PNI. Model 3: adjusted for age, sex, ECOG, radical resection, TNM stage, KPS, physical activity, nutritional intervention, postoperative chemoradiotherapy, lymphocytes, neutrophils, WBC, AST, ALT, serum albumin, PNI, family history of cancer, tea consumption, alcohol consumption, smoking, diabetes, hypertension, coronary heart disease, platelet, haemoglobin, and serum total protein.

### Stratified analyses by potential effect modifiers

**Figure 5:**
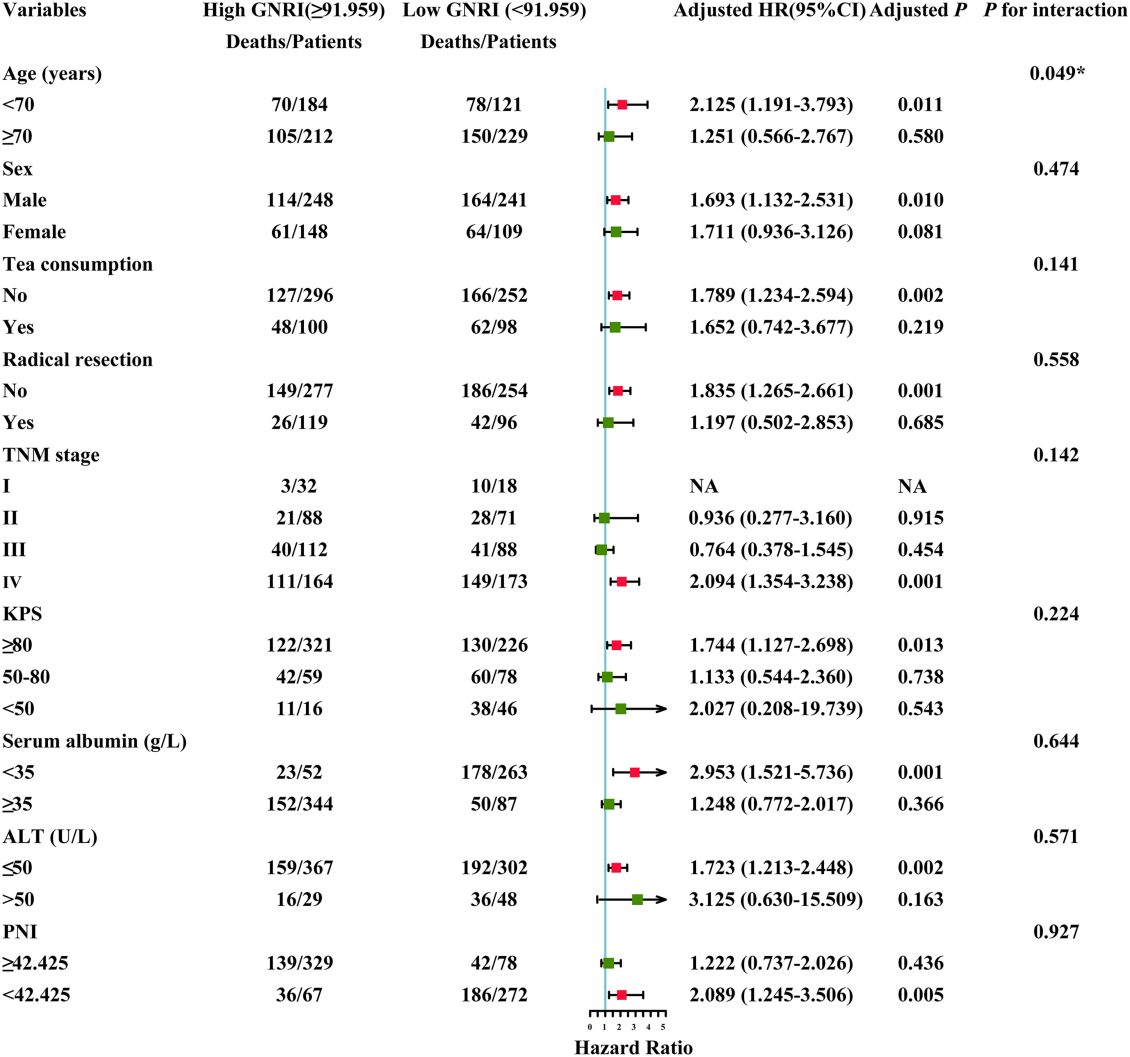
The stratification analysis of GNRI and the OS of EPCC. ALT, alanine transaminase; CI, confidence interval; EPCC, Elderly Patients with Cancer Cachexia; GNRI, Geriatric Nutritional Risk Index; HR, hazard ratio; KPS, Karnofsky Performance Status; OS, overall survival; PNI, Prognostic Nutritional Index; TNM stage, tumour‐node‐metastasis stage.

The stratified analysis parameters were selected according to the results of multivariate survival analysis. A significantly stronger positive association between GNRI (low GNRI, <91.959 vs. high GNRI, ≥91.959) and death risk of EPCC was observed in patients with aged <70 years (*P* for interaction = 0.049). Additionally, when compared with patients with high GNRI, the patients with low GNRI were associated with the increased death risk of EPCC, which could be observed in patients with aged < 70 years [*P* = 0.011, HR (95% CI): 2.125 (1.191–3.793)], male patients [*P* = 0.010, HR (95% CI): 1.693 (1.132–2.531)], patients without tea consumption habit [*P* = 0.002, HR (95% CI): 1.789 (1.234–2.594)], patients without radical resection [*P* = 0.001, HR (95% CI): 1.835 (1.265–2.661)], patients with stage IV [*P* = 0.001, HR (95% CI): 2.094 (1.354–3.238)], KPS ≥ 80 [*P* = 0.013, HR (95% CI): 1.744 (1.127–2.698)], serum albumin <35 g/L [*P* = 0.001, HR (95% CI): 2.953 (1.521–5.736)], ALT ≤50 U/L [*P* = 0.002, HR (95% CI): 1.723 (1.213–2.448)], and PNI < 42.425[*P* = 0.005, HR (95% CI): 2.089 (1.245–3.506)] (*Figure* 5).

### Constructed risk scored model and sensitive analysis

**Figure 6:**
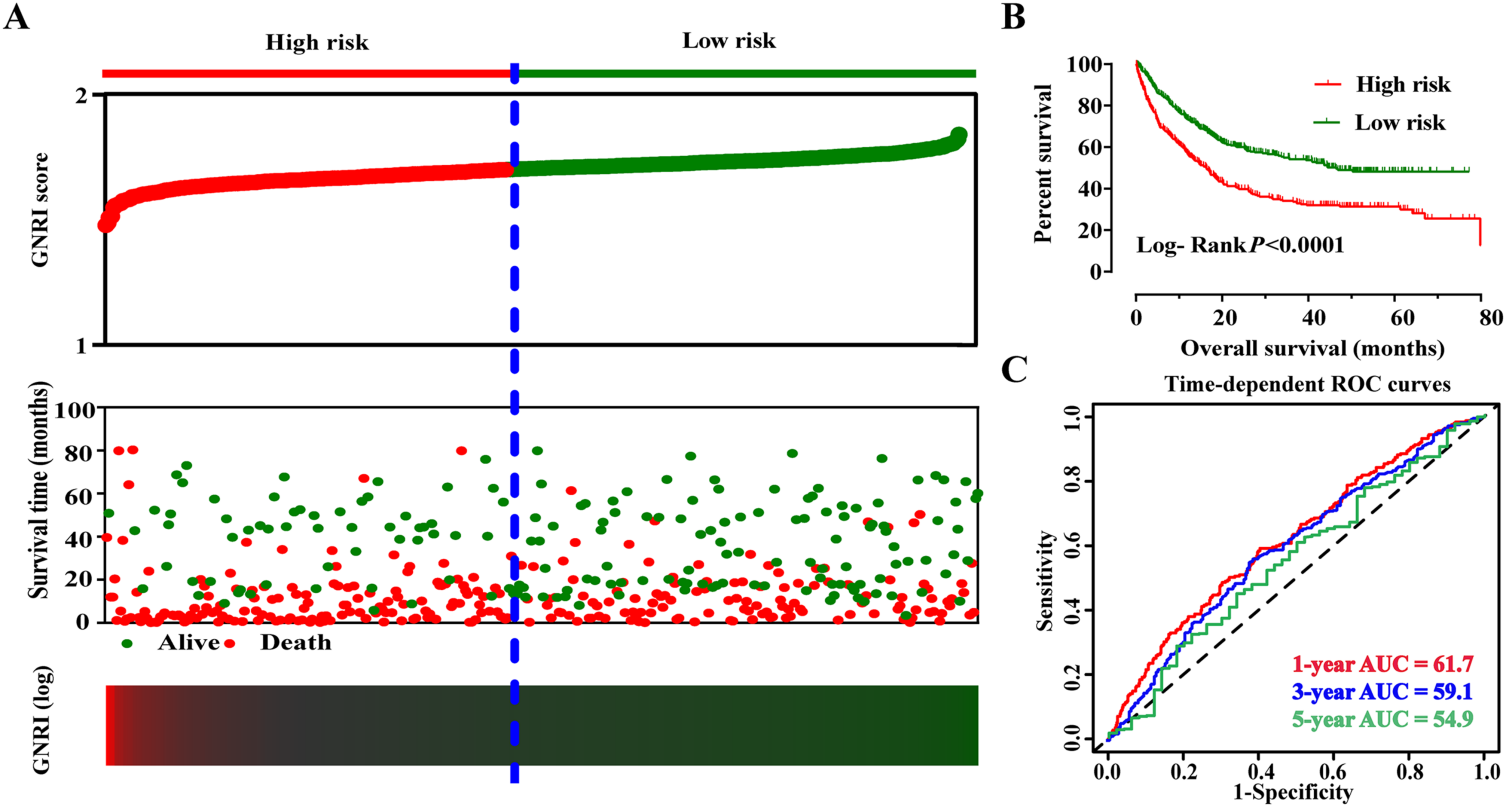
The prognostic risk score model of EPCC based on GNRI score. AUC, area under curve; EPCC, Elderly Patients with Cancer Cachexia; GNRI, Geriatric Nutritional Risk Index.

We constructed a prognostic risk model based on the GNRI score. The risk score was based on the GNRI score and β regression risk coefficient. The β coefficient was obtained according to the multivariate cox regression risk model. Risk score = GNRI score × 0.578. The higher the GNRI score obtained by a patient, and the greater the risk score, the lower the patient's the prognostic risk. The cut‐off value of the risk score was based on the GNRI score. Additionally, a prognostic heat map, survival curve, and time‐dependent survival curve were performed based on the risk score. The results showed that patients with a high risk of the score had a worse survival than those with a low‐risk score. The prognostic ROC showed that the 1, 3, and 5 year AUCs were 61.7, 59.1, and 54.9, respectively (*Figure* 6).

Considering that GNRI was a prognostic indicator for EPCC, we performed a sensitivity analysis after excluding information on patients who died within three months. The cut‐off value was consistent with the previous description. The sensitivity analysis showed that GNRI was still an independent prognostic indicator for elderly patients with cachexia. Additionally, the sensitivity analysis of different types of cancers was basically in correspondence with previous results (Supporting Information, *Table* S2).

## Discussion

Cancer cachexia is very common in elderly patients with cancer and can be diagnosed by unintentional weight loss, BMI, and skeletal muscle exhaustion.^4^, ^6^ However, there are few studies related to the clinical outcome of EPCC. As far as we know, we first investigated the clinical prognostic value of the GRNI score in EPCC. In this cohort study, we found that 26.3% of cancer patients were cancer cachexia patients. Among patients with cancer cachexia, EPCC accounted for approximately 29.1% of all patients with cancer cachexia. Additionally, we also assessed that the GNRI scores in patients with cancer cachexia and EPCC were significantly higher than those in patients with non‐cancer cachexia and young patients with cancer cachexia. That is to say, patients with cancer cachexia, especially EPCC, will face a high risk of malnutrition and a worse prognosis.

The calibration curves suggested that GNRI scores showed good predictive ability in 1, 3, and 5 year survival outcomes of OS in EPCC. Our survival analysis results showed that the low GNRI score of EPCC had a worse survival prognosis and shorter OS than those with a high GNRI score. After adjusting by different multivariate adjustment models, the GNRI could predict the OS of EPCC, whether as a continuous variable or a categorical variable. Particularly, the optional cut‐off value of GNRI in our study, the low GNRI still showed statistical significance and increased the death risk of OS in EPCC. The prognostic risk model we constructed also showed an excellent prognostic prediction effect. Additionally, our sensitivity analysis also found similar results. Interestingly, our subgroup analysis found that aging was related to GNRI score in older patients with cancer cachexia. In short, GNRI may be an independent prognostic factor for EPCC, especially in older patients with cancer cachexia of GC and CRC.

Our results found that GNRI can predict the short‐term and long‐term clinical outcomes of EPCC. Previous reports also found the prognostic value of GNRI score in elderly patients with malignant tumours. A randomized controlled trial by Lee *et al*. found that GNRI score was an independent prognostic factor for OS in elderly patients with extensive‐stage disease small cell lung cancer (*P* = 0.020, HR: 1.539; 95% CI: 1.069–2.216).^19^ A study, which enrolled in 739 patients with primary lung cancer who underwent surgery, by Hino *et al*. found GNRI could improve the predicting ability of survival after lung cancer surgery.^27^ A similar result was also found in the studies by Shoji *et al*.^15^ The GNRI can also predict the OS of geriatric patients with metastatic lung adenocarcinoma.^28^ The same effect has been found in malignant tumours of the digestive system. The study by Yamana *et al*. showed that the GNRI could assess the nutritional status and predict the complications in patients with esophageal cancer.^29^ Wang *et al*.'s study suggested that the GNRI independent prognostic factors for OS and progression‐free survival (PFS). The GNRI could also help in the risk stratification of elderly patients undergoing Radiotherapy (RT) or definitive concurrent chemoradiotherapy (dCRT).^30^ Similar results of GNRI also were found in predicting the postoperative survival in Elderly Esophageal Squamous Cell Carcinoma (ESCC)^23^ and these patients with Radiotherapy.^31^ As for related reports on GC and CRC, a study by Hirahara *et al*. found that preoperative GNRI could predict the short‐ and long‐term outcomes of OS in elderly patients with GC.^32^ Kushiyama *et al*. and Sugawara *et al*. also found the same result of GNRI for OS in GC.^25^, ^33^ The study by Tang *et al*. showed that the GNRI is an effective tool for predicting the long‐term prognosis and provides a scientific basis for early nutrition interventions of elderly CRC patients.^24^ Sasaki *et al*.'s study suggested that the preoperative GNRI can be used as an identifier for the potential morbidity and mortality of elderly CRC patients. Interestingly, the GNRI is also a prognostic factor for patients with colorectal liver metastasis.^34^ However, similar results have been found in other malignancies.^35^ In general, GNRI can predict the survival, postoperative complications, tumour metastasis, and recurrence of elderly patients with cancer.

The GNRI is composed of the serum albumin level and body weight. Regarding weight loss, GNRI seems to indicate the severity of the systemic disease and the protein‐calorie storage that the patient needs to cope with acute stress.^35^ However, the weighted value of serum albumin is more significant than bodyweight in the GNRI.^14^ The serum albumin is a known indicator of inflammatory microenvironment^36^ or patients with cancer cachexia. The body of patients with cancer is in a systemic inflammatory state as the disease progresses. Increased circulating concentrations of various inflammatory cytokines have been found in patients with cancer cachexia.^37^ Systemic inflammation is the main cause of malnutrition and patients with cancer cachexia. Additionally, the systemic inflammatory response is closely related to tumour progression and metastasis.^38^ Hypoalbuminaemia is a systemic inflammatory response. Under inflammatory conditions, inflammatory cytokines such as IL‐6 reduce liver albumin synthesis and its mRNA content.^39^ IL‐2 can promote the escape of albumin, and oxidative stress can cause albumin degeneration.^40^ The previous study has described the potential function of GNRI in systemic inflammation and cachexia^19^; furthermore, the associated factors can also be reflecting the body's nutritional status, immune status, and inflammation status. In other words, hypoalbuminaemia can reflect the severity of the disease and the protein‐energy imbalance in patients with cancer cachexia.^37^ Therefore, GNRI combined with serum albumin and body weight may be a better predictor of patients with cancer cachexia than serum albumin alone.

This negative energy balance is caused by insufficient feeding caused by anorexia and metabolic factors such as insulin resistance and excessive muscle protein catabolism.^41^ Anorexia is affected by hormones through neuropeptide networks but also by the mechanical effects of tumours and the pro‐inflammatory environment that produces high metabolism.^42^ The initial manifestation of sarcopenia is the loss of skeletal muscle mass and strength due to aging. Skeletal muscle exhaustion is a key feature of cancer cachexia, and its consequences include increased chemotherapy toxicity, cancer surgery complications, and mortality.^43^ In the elderly patient population, these characteristics of cachexia may be amplified or concealed, and the consequences will be even less optimistic. In the real world, elderly patients usually have poor nutritional status and are prone to cancer‐related deaths. The reduction of albumin metabolic reserve due to age may often cause patients to fail to cope with the systemic inflammatory pressure caused by cachexia. On the other hand, advanced age is associated with poor adaptation to disease‐related metabolic stress. It is considered an independent predictor of poor clinical outcome.^41^ Therefore, compared with other single parameters, GRNI, which can reflect the patient's weight, muscle loss, and systemic inflammation may be a better surrogate indicator for assessing the severity of EPCC.

Indeed, our research still has some limitations. First, this study is a multi‐centre retrospective study, there will be some influences of selection and information bias, and further well‐designed prospective trials need to be verified. Second, this is the prognostic impact of a single indicator, and more indicators (including related inflammatory and nutritional indicators such as IL‐6, CRP, and protein and calorie intake) may be added for a comprehensive evaluation in the future. Third, this finding requires cohort verification in a larger sample, multiple regions, multiple countries, and different ethnic groups.

## Conclusions

In conclusion, this study, for the first time, found that the GNRI score is a new, potential, and independent predictive prognostic factor for EPCC. GNRI can predict the short‐term and long‐term clinical outcomes of EPCC. Compared with patients with high scores of GNRI (≥91.959), low scores of GNRI (<91.959) increase the risk of death and reduce OS in EPCC. However, this result needs further confirmation.

## Funding

This work was supported by the National Key Research and Development Program (2017YFC1309200).

## Conflict of interest

The authors declare that there is no conflict of interest.

## Ethics statement

This study followed the Helsinki declaration. All participants signed an informed consent form and this study was approved by the Institutional Review Board of each hospital (Registration number: ChiCTR1800020329).

## Supporting information


**Figure S1** Flowchart of patient selection for this study.

**Figure S2** Optimal cut‐off value of GNRI and PNI according to ROC curve.

**Table S1** Univariate and multivariate analyses of OS in cancer patients.

**Table S2** Sensitivity analysis on the OS of GNRI in overall cancer patients and different types of cancer patients.
