# Supplementary figures and images for: Geriatric Nutrition Risk Index: Prognostic factor related to inflammation in elderly patients with cancer cachexia
Source: J Cachexia Sarcopenia Muscle. 2021 Sep 29;12(6):1969–82. doi: 10.1002/jcsm.12800 (PMC8718015; doi:10.1002/jcsm.12800)

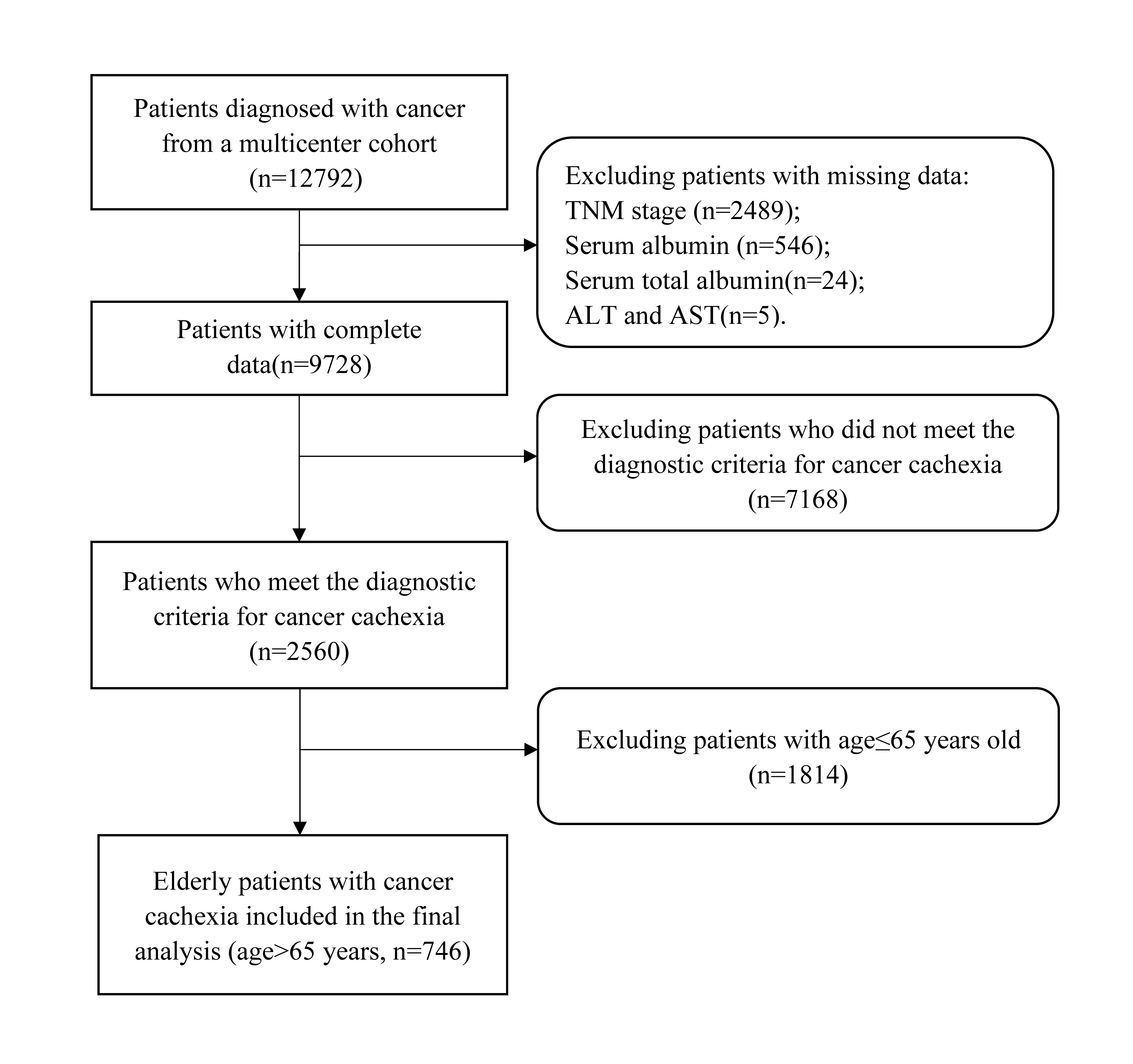

Supplement: Supplementary file 1 — Figure S1 Flowchart of patient selection for this study. [file JCSM-12-1969-s003.tif]

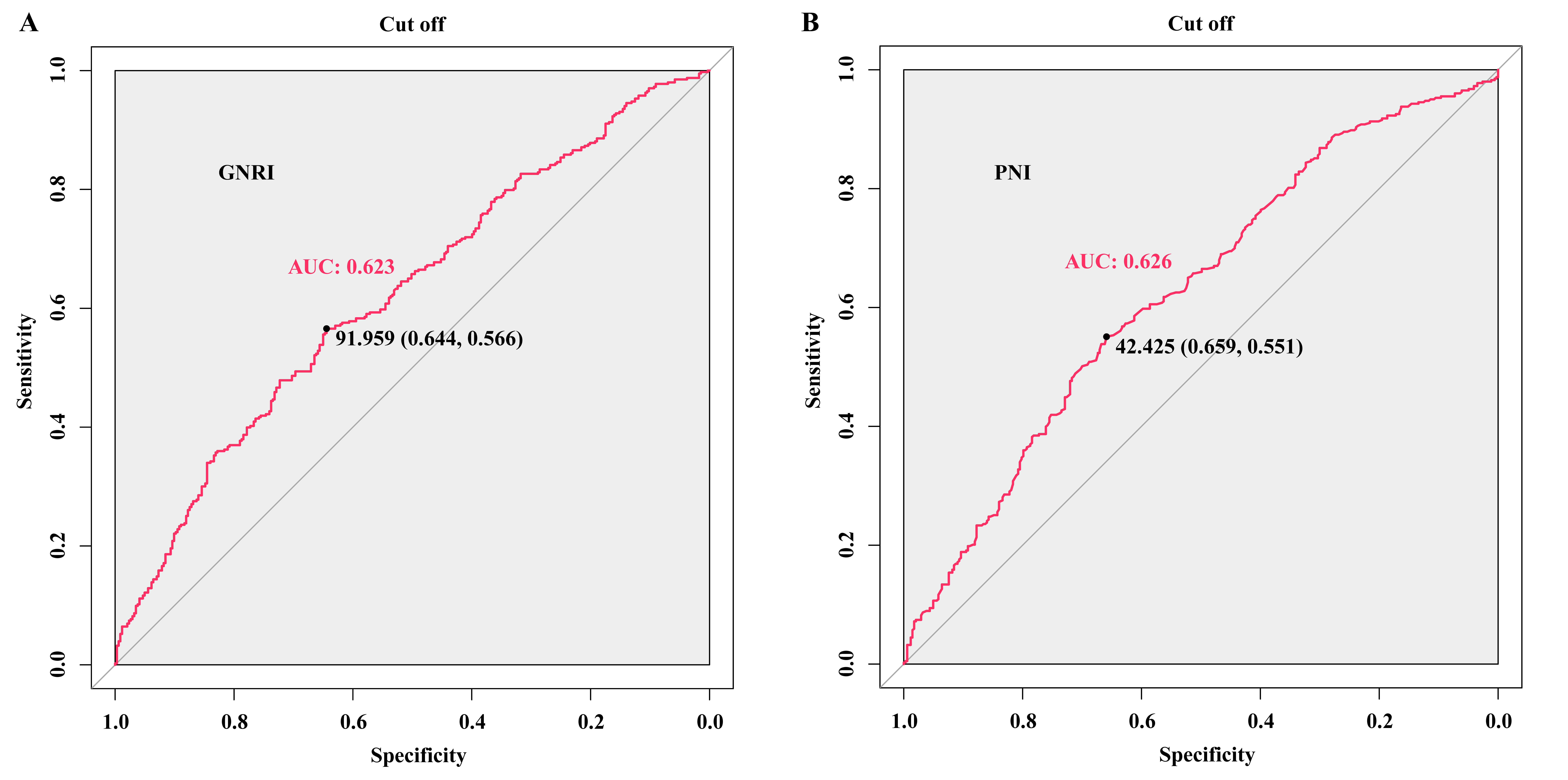

Supplement: Supplementary file 2 — Figure S2 Optimal cut‐off value of GNRI and PNI according to ROC curve. [file JCSM-12-1969-s004.tif]
