# Supplementary material for: Geriatric Nutrition Risk Index: Prognostic factor related to inflammation in elderly patients with cancer cachexia
Source: J Cachexia Sarcopenia Muscle. 2021 Sep 29;12(6):1969–82. doi: 10.1002/jcsm.12800 (PMC8718015; doi:10.1002/jcsm.12800)
Supplement: Supplementary file 3 — Table S1 Univariate and multivariate analyses of OS in cancer patients. [file JCSM-12-1969-s001.docx]

**Table S1 Univariate and multivariate analyses of OS in cancer patients**

|  | OS | |  | OS* | |
| --- | --- | --- | --- | --- | --- |
| Variables | Crude HR (95%CI) | Crude *P* |  | Adjusted HR (95%CI) | Adjusted *P* |
| Age (year) |  |  |  |  |  |
| <70 | 1 |  |  | 1 |  |
| ≥70 | 1.252 (1.022-1.534) | 0.030 |  | 1.014 (0.815-1.26) | 0.903 |
| Sex |  |  |  |  |  |
| Male | 1 |  |  | 1 |  |
| Female | 0.794 (0.642-0.981) | 0.032 |  | 0.804 (0.605-1.068) | 0.132 |
| Tea consumption |  |  |  |  |  |
| No | 1 |  |  | 1 |  |
| Yes | 1.017 (0.816-1.267) | 0.880 |  | 0.762 (0.593-0.978) | 0.032 |
| Alcohol consumption |  |  |  |  |  |
| No | 1 |  |  | 1 |  |
| Yes | 0.971 (0.765-1.232) | 0.807 |  | 1.104 (0.835-1.459) | 0.486 |
| Smoking |  |  |  |  |  |
| No | 1 |  |  | 1 |  |
| Yes | 1.128 (0.927-1.372) | 0.229 |  | 1.106 (0.844-1.450) | 0.464 |
| Family history of cancer |  |  |  |  |  |
| No | 1 |  |  | 1 |  |
| Yes | 1.185 (0.893-1.571) | 0.240 |  | 1.300 (0.965-1.752) | 0.084 |
| Diabetes |  |  |  |  |  |
| No | 1 |  |  | 1 |  |
| Yes | 1.044 (0.782-1.394) | 0.770 |  | 1.160 (0.855-1.573) | 0.342 |
| Hypertension |  |  |  |  |  |
| No | 1 |  |  | 1 |  |
| Yes | 1.151 (0.925-1.433) | 0.207 |  | 1.078 (0.848-1.37) | 0.541 |
| Coronary heart disease |  |  |  |  |  |
| No | 1 |  |  | 1 |  |
| Yes | 0.937 (0.668-1.314) | 0.706 |  | 0.844 (0.591-1.204) | 0.349 |
| BMI (kg/m^2) |  |  |  |  |  |
| <18.5 | 1 |  |  | 1 |  |
| 18.5-24 | 0.766 (0.617-0.951) | 0.016 |  | 1.062 (0.818-1.378) | 0.652 |
| >24 | 0.634 (0.457-0.878) | 0.006 |  | 1.469 (0.967-2.232) | 0.072 |
| ECOG PS, n (%) |  |  |  |  |  |
| <2 | 1 |  |  | 1 |  |
| ≥2 | 1.933 (1.582-2.363) | <0.001 |  | 1.102 (0.861-1.409) | 0.441 |
| Postoperative chemoradiotherapy | |  |  |  |  |
| No | 1 |  |  | 1 |  |
| Yes | 1.528 (1.256-1.858) | <0.001 |  | 1.105 (0.885-1.380) | 0.379 |
| Radical resection |  |  |  |  |  |
| No | 1 |  |  | 1 |  |
| Yes | 0.318 (0.245-0.414) | <0.001 |  | 0.563 (0.397-0.798) | 0.001 |
| TNM stage |  |  |  |  |  |
| Ⅰ | 1 |  |  | 1 |  |
| Ⅱ | 1.371 (0.743-2.527) | 0.313 |  | 1.335 (0.718-2.486) | 0.361 |
| Ⅲ | 2.001 (1.113-3.595) | 0.020 |  | 1.856 (1.018-3.384) | 0.044 |
| Ⅳ | 6.477 (3.699-11.34) | <0.001 |  | 4.580 (2.455-8.541) | <0.001 |
| KPS |  |  |  |  |  |
| ≥80 | 1 |  |  | 1 |  |
| 50-80 | 2.278 (1.808-2.869) | <0.001 |  | 1.673 (1.265-2.213) | <0.001 |
| <50 | 2.259 (1.654-3.084) | <0.001 |  | 1.873 (1.156-3.036) | 0.011 |
| Physical activity |  |  |  |  |  |
| Normal | 1 |  |  | 1 |  |
| Limited | 1.606 (1.298-1.989) | <0.001 |  | 1.228 (0.942-1.599) | 0.129 |
| Inactivity | 1.969 (1.362-2.847) | <0.001 |  | 0.809 (0.466-1.406) | 0.453 |
| Nutritional intervention |  |  |  |  |  |
| No | 1 |  |  | 1 |  |
| Yes | 0.886 (0.700-1.122) | 0.315 |  | 0.828 (0.648-1.060) | 0.135 |
| Serum total protein (g/L) |  |  |  |  |  |
| <60 | 1 |  |  | 1 |  |
| ≥60 | 0.881 (0.707-1.098) | 0.260 |  | 1.203 (0.915-1.582) | 0.185 |
| Serum albumin (g/L) |  |  |  |  |  |
| <35 | 1 |  |  | 1 |  |
| ≥35 | 0.629 (0.517-0.765) | <0.001 |  | 1.407 (1.022-1.937) | 0.036 |
| AST (U/L) |  |  |  |  |  |
| ≤40 | 1 |  |  | 1 |  |
| >40 | 1.446 (1.126-1.857) | 0.004 |  | 0.933 (0.656-1.326) | 0.697 |
| ALT (U/L) |  |  |  |  |  |
| ≤50 | 1 |  |  | 1 |  |
| >50 | 1.648 (1.231-2.207) | 0.001 |  | 1.808 (1.195-2.736) | 0.005 |
| Hemoglobin (g/L) |  |  |  |  |  |
| Male<110 and Female<120 | 1 |  |  | 1 |  |
| Male≥110 and Female≥120 | 0.832 (0.684-1.012) | 0.066 |  | 1.021 (0.81-1.289) | 0.858 |
| WBC (×10^9/L) |  |  |  |  |  |
| ≤10 | 1 |  |  | 1 |  |
| >10 | 1.760 (1.384-2.238) | <0.001 |  | 1.276 (0.728-2.238) | 0.395 |
| Neutrophils (×10^9/L) |  |  |  |  |  |
| ≤8 | 1 |  |  | 1 |  |
| >8 | 2.091 (1.633-2.679) | <0.001 |  | 1.173 (0.661-2.082) | 0.973 |
| Lymphocytes (×10^9/L) |  |  |  |  |  |
| ≤1.5 | 1 |  |  | 1 |  |
| >1.5 | 0.729 (0.592-0.898) | 0.003 |  | 1.005 (0.774-1.304) | 0.132 |
| Platelet (×10^9/L) |  |  |  |  |  |
| ≤160 | 1 |  |  | 1 |  |
| >160 | 0.985 (0.783-1.239) | 0.896 |  | 0.932 (0.733-1.186) | 0.568 |
| PNI |  |  |  |  |  |
| ≥42.425 | 1 |  |  | 1 |  |
| <42.425 | 1.772 (1.455-2.158) | <0.001 |  | 1.687 (1.181-2.411) | 0.004 |

Notes: OS*: Adjusted model 3 for GNRI OS: All variables. OS, Overall Survival; HR, Hazards Ratio; CI, Confidence Interval; BMI: Body Mass Index; ECOG PS: Eastern Cooperative Oncology Group Performance Status; KPS, Karnofsky Performance Status; AST: Aspertate Aminotransferase; ALT: Alanine Transaminase; WBC: White Blood Cells; PNI: Prognostic Nutritional Index; GNRI: Geriatric Nutritional Risk Index.
