# Supplementary material for: Geriatric Nutrition Risk Index: Prognostic factor related to inflammation in elderly patients with cancer cachexia
Source: J Cachexia Sarcopenia Muscle. 2021 Sep 29;12(6):1969–82. doi: 10.1002/jcsm.12800 (PMC8718015; doi:10.1002/jcsm.12800)
Supplement: Supplementary file 4 — Table S2 Sensitivity analysis on the OS of GNRI in overall cancer patients and different types of cancer patients. [file JCSM-12-1969-s002.docx]

**Table S2 Sensitivity analysis on the OS of GNRI in overall cancer patients and different types of cancer patients**

| Variables | OS | |  | OS* | |
| --- | --- | --- | --- | --- | --- |
|  | Unadjusted HR (95%CI) | Unadjusted *P* |  | Adjusted HR (95%CI) | Adjusted *P* |
| Overall patients |  |  |  |  |  |
| As continuous (per SD) | 0.804 (0.726-0.891) | <0.001 |  | 0.747 (0.610-0.915) | 0.005 |
| By GNRI cut-off |  |  |  |  |  |
| GNRI ≥91.59 | 1 |  |  | 1 |  |
| GNRI <91.59 | 1.583 (1.266-1.978) | <0.001 |  | 1.727 (1.193-2.500) | 0.004 |
| By GNRI cut-off |  |  |  |  |  |
| GNRI <91.959 | 1 |  |  | 1 |  |
| GNRI ≥91.959 | 0.632 (0.505-0.790) | <0.001 |  | 0.579 (0.400-0.838) | 0.004 |
| By Interquartile |  |  |  |  |  |
| Q1 (99.557~) | 1 |  |  | 1 |  |
| Q2 (92.922~99.557) | 1.144 (0.827-1.584) | 0.416 |  | 1.114 (0.758-1.638) | 0.582 |
| Q3 (84.521~92.922) | 1.588 (1.190-2.120) | 0.002 |  | 1.861 (1.169-2.961) | 0.009 |
| Q4 (~84.521) | 1.788 (1.284-2.489) | 0.001 |  | 2.252 (1.18-4.298) | 0.014 |
| *P* for trends |  | <0.001 |  |  | 0.007 |
| By tumor types |  |  |  |  |  |
| Lung cancer |  |  |  |  |  |
| GNRI ≥91.59 | 1 |  |  | 1 |  |
| GNRI <91.59 | 1.624 (1.075-2.452) | 0.021 |  | 1.251 (0.566-2.767) | 0.580 |
| Gastrointestinal cancer |  |  |  |  |  |
| Gastric cancer |  |  |  |  |  |
| GNRI ≥91.59 | 1 |  |  | 1 |  |
| GNRI <91.59 | 1.330 (0.811-2.181) | 0.259 |  | 2.155 (0.911-5.103) | 0.081 |
| Colorectal cancer |  |  |  |  |  |
| GNRI ≥91.59 | 1 |  |  | 1 |  |
| GNRI <91.59 | 1.547 (0.943-2.538) | 0.084 |  | 3.115 (1.210-8.018) | 0.018 |
| Esophageal cancer |  |  |  |  |  |
| GNRI ≥91.59 | 1 |  |  | 1 |  |
| GNRI <91.59 | 2.445 (1.250-4.784) | 0.009 |  | 13.901 (1.634-118.266) | 0.016 |
| Other gastrointestinal  cancer | |  |  |  |  |
| GNRI ≥91.59 | 1 |  |  | 1 |  |
| GNRI <91.59 | 0.716 (0.310-1.652) | 0.443 |  | NA | NA |
| Other cancer subtypes |  |  |  |  |  |
| GNRI ≥91.59 | 1 |  |  | 1 |  |
| GNRI <91.59 | 2.470 (1.229-4.963) | 0.011 |  | 29.370 (1.405-613.887) | 0.029 |

Notes: The sensitivity analysis was to exclude patients who died within **3** months. *: Adjusted for Age, Sex, ECOG, Radical resection, TNM stage, KPS, **Physical activity,** **Nutritional intervention,** Postoperative chemoradiotherapy, Lymphocytes, Neutrophils, WBC, AST, ALT, Serum albumin, PNI, Family history of cancer, Tea consumption, Alcohol consumption, Smoking, Diabetes, Hypertension, Coronary heart disease, Platelet, Hemoglobin, Serum total protein. OS, Overall Survival; HR, Hazards Ratio; CI, Confidence Interval; BMI: Body Mass Index; ECOG PS: Eastern Cooperative Oncology Group Performance Status; KPS, Karnofsky Performance Status; AST: Aspertate Aminotransferase; ALT: Alanine Transaminase; WBC: White Blood Cells; PNI: Prognostic Nutritional Index**; GNRI: Geriatric Nutritional Risk Index.**
